# Inflammation–Matrix Crosstalk in Vesicoureteral Reflux: Serum and Urinary ECM-Related Biomarkers and Renal Scarring

**DOI:** 10.3390/ijms27114815

**Published:** 2026-05-27

**Authors:** Marija Ratković-Janković, Jelena Bašić, Tatjana Jevtović-Stoimenov, Emilija Golubović, Ljiljana Pejčić, Ivana Nikolić, Bojko Bjelaković

**Affiliations:** 1 Department of Pediatrics, Faculty of Medicine, University of Niš, 18000 Niš, Serbia; emilija.golubovic@medfak.ni.ac.rs (E.G.); ljiljana.pejcic@medfak.ni.ac.rs (L.P.); bojko.bjelakovic@medfak.ni.ac.rs (B.B.); 2 Clinic of Pediatrics, University Clinical Center Niš, 18000 Niš, Serbia; 3 Department of Biochemistry, Faculty of Medicine, University of Niš, 18000 Niš, Serbia; jelena.basic@medfak.ni.ac.rs (J.B.); tatjana.jevtovic.stoimenov@medfak.ni.ac.rs (T.J.-S.); 4 Gynecology and Obstetrics Clinic “Narodni Front”, 11000 Belgrade, Serbia; nikolic.ivana@gakfront.org

**Keywords:** vesicoureteral reflux, extracellular matrix, MMP-9, TIMP-1, CD147, MCP-1, TGF-β, renal scarring

## Abstract

This study evaluated serum and urinary extracellular matrix (ECM)-related biomarkers in pediatric patients with vesicoureteral reflux (VUR) and their association with disease severity and renal scarring. Children with VUR and control subjects were included. Serum and urinary concentrations of matrix metalloproteinase-9 (MMP-9), tissue inhibitor of metalloproteinases-1 (TIMP-1), CD147, transforming growth factor-β (TGF-β), and monocyte chemoattractant protein-1 (MCP-1) were analyzed, with urinary values normalized to creatinine. Serum MCP-1 levels were higher in patients with VUR (*p* < 0.001), whereas other serum biomarkers showed no significant differences. Creatinine-normalized urinary TIMP-1 and TGF-β levels were significantly elevated (*p* = 0.006 and *p* = 0.008, respectively), while CD147 levels were reduced (*p* = 0.011) in VUR patients. Receiver operating characteristic analysis demonstrated moderate discriminative performance for uTIMP-1/Cr (AUC = 0.74), uTGF-β/Cr (AUC = 0.73), and uCD147/Cr (AUC = 0.72). Higher urinary levels of MMP-9 (*p* = 0.014), TIMP-1 (*p* = 0.003), and MCP-1 (*p* = 0.015) were associated with high-grade VUR and with bilateral renal scarring (*p* = 0.048, *p* = 0.041, and *p* = 0.025, respectively). These findings suggest that urinary ECM-related biomarkers may reflect intrarenal inflammatory and fibrotic processes in VUR. However, their clinical applicability remains limited and requires validation in larger, longitudinal studies.

## 1. Introduction

Vesicoureteral reflux (VUR) is one of the most common congenital anomalies of the urinary tract in childhood and represents a significant risk factor for recurrent urinary tract infections (UTIs) and the development of reflux nephropathy (RN) [1,2,3]. The long-term consequences of VUR-associated renal damage include permanent renal scarring, arterial hypertension, proteinuria, and progression to chronic kidney disease (CKD) [2,4]. Importantly, the presence and extent of renal scarring do not always correlate with the grade of reflux, suggesting a crucial role of host inflammatory and molecular mechanisms in determining renal outcomes [3,5].

The contemporary concept of reflux nephropathy pathogenesis considers renal scarring as an active and dynamic biological process rather than merely a mechanical consequence of retrograde urine flow [5]. Recurrent febrile UTIs and episodes of sterile reflux may induce a persistent inflammatory response within the renal parenchyma, leading to tubular epithelial injury, interstitial leukocyte infiltration, and activation of profibrotic signaling pathways [3]. A central role in these processes is played by the interaction between inflammation and extracellular matrix (ECM) remodeling, known as inflammation–matrix crosstalk [4].

The extracellular matrix is no longer regarded as a passive structural scaffold but as a dynamic and bioactive compartment involved in regulating cell adhesion, migration, immune cell recruitment, and cytokine signaling [4,6,7]. Under physiological conditions, ECM homeostasis is maintained by a finely balanced interplay between its synthesis and degradation, primarily regulated by matrix metalloproteinases (MMPs) and their endogenous inhibitors, tissue inhibitors of metalloproteinases (TIMPs) [6]. Chronic inflammation disrupts this balance, favoring excessive ECM deposition, myofibroblast activation, and progressive fibrotic remodeling, which represent key mechanisms underlying renal scarring and CKD progression [4,6,8].

In the context of VUR, persistent inflammatory stimuli promote the release of chemokines and cytokines that enhance inflammatory cell recruitment and further stimulate ECM remodeling [9]. Monocyte chemoattractant protein-1 (MCP-1/CCL2) has been recognized as a key mediator of monocyte–macrophage infiltration and interstitial inflammation in reflux nephropathy [4,6,10]. Transforming growth factor beta (TGF-β), a central profibrotic cytokine, promotes fibroblast activation, epithelial-to-mesenchymal transition, and collagen accumulation [4,11,12]. In parallel, dysregulation of MMP and TIMP activity contributes to impaired ECM turnover and matrix accumulation [6].

It is important to emphasize that numerous inflammatory and ECM-related mediators can be detected in both serum and urine, potentially reflecting ongoing intrarenal pathological processes [9,13]. Recent studies suggest that urinary biomarkers, including MCP-1, NGAL, MMP-9, and TIMP-1, may correlate with inflammation and renal scarring in children with VUR [9,10,14]. These biomarkers may offer advantages over conventional diagnostic methods, such as DMSA scintigraphy, which detects scarring only after irreversible structural damage has occurred. Therefore, serum and urinary ECM-related biomarkers may provide a non-invasive insight into active inflammatory and fibrotic processes and enable earlier identification of patients at increased risk for progressive renal injury.

This study aimed to investigate the association of serum and urinary ECM-related inflammatory mediators with renal scarring and disease severity in children with VUR.

## 2. Results

### 2.1. Demographic and Clinical Characteristics According to Study Groups

The study included 72 patients with vesicoureteral reflux (VUR) and 21 subjects in the control group. Patients with VUR were significantly younger than controls (*p* < 0.001). Significant differences between the VUR and control groups were observed for C-reactive protein (CRP) (*p* = 0.001), serum urea (*p* = 0.006), and urinary creatinine (*p* = 0.006). Despite the observed between-group differences, the parameter levels remained within the reference ranges in both the VUR and control groups. No significant differences were found for serum creatinine (*p* = 0.298) or urinary microalbumin (*p* = 0.777). Sex distribution did not differ significantly between the groups (*p* = 1.000; Table 1).

### 2.2. Serum and Urinary Biomarker Levels in the Study Groups

Serum levels of MMP-9, TIMP-1, CD147, and TGF-β did not differ significantly between patients with VUR and control subjects (*p* > 0.05). In contrast, serum MCP-1 levels were significantly higher in patients with VUR compared with controls (*p* < 0.001). Urinary concentrations of MMP-9, TIMP-1, TGF-β, and MCP-1 did not differ significantly between the study groups. Urinary CD147 levels were significantly lower in patients with VUR than in the control group (*p* < 0.001). After normalization to urinary creatinine, urinary TIMP-1 (***p*** = 0.006) and TGF-β levels (*p* = 0.008) were significantly higher in patients with VUR than in controls, whereas CD147 levels were significantly lower in the VUR group (*p* = 0.011). All significant findings in the primary analysis remained significant after false discovery rate (FDR) correction. Although MMP-9 and MCP-1 levels tended to be higher in patients with VUR, the differences between the groups did not reach statistical significance (*p* > 0.05; Table 2).

To account for potential confounding factors, multivariate logistic regression analysis was performed, adjusting for age and sex. In the adjusted models, none of the investigated urinary biomarkers remained independently associated with VUR. Specifically, uMMP-9/Cr (OR = 1.004, 95% CI: 0.997–1.011, *p* = 0.297), uTIMP-1/Cr (OR = 1.003, 95% CI: 0.997–1.010, *p* = 0.310), uTGF-β/Cr (OR = 1.011, 95% CI: 0.995–1.028, *p* = 0.184) and uCD147/Cr (OR = 0.9997, 95% CI: 0.9992–1.0001, *p* = 0.122) did not show independent associations after adjustment. Age remained significantly associated with VUR (*p* = 0.014), whereas sex was not a significant predictor (Table 3).

### 2.3. Receiver Operating Characteristic (ROC) Curve Analysis

**Figure 1:**
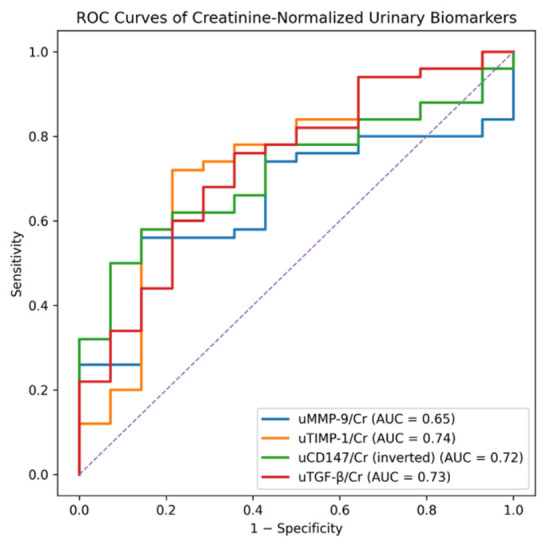
Receiver operating characteristic (ROC) curves of creatinine-normalized urinary biomarkers for discrimination between patients with vesicoureteral reflux (VUR) and controls.

ROC curve analysis demonstrated that uTIMP-1/Cr and uTGF-β/Cr showed moderate diagnostic accuracy for discriminating patients with VUR from controls, with AUCs of 0.74 (95% CI: 0.61–0.87; *p* < 0.001) and 0.73 (95% CI: 0.60–0.87; *p* < 0.001), respectively. After inversion to account for lower concentrations in VUR, uCD147/Cr also exhibited moderate discriminative performance (AUC = 0.72, 95% CI: 0.58–0.86; *p* = 0.002). In contrast, uMMP-9/Cr showed poor diagnostic accuracy with borderline statistical significance (AUC = 0.65, 95% CI: 0.49–0.80; *p* = 0.058; Figure 1). Detailed diagnostic performance parameters are provided in Supplementary Table S1.

### 2.4. Serum and Urinary Biomarker Levels in Patients According to VUR Grade

**Figure 2:**
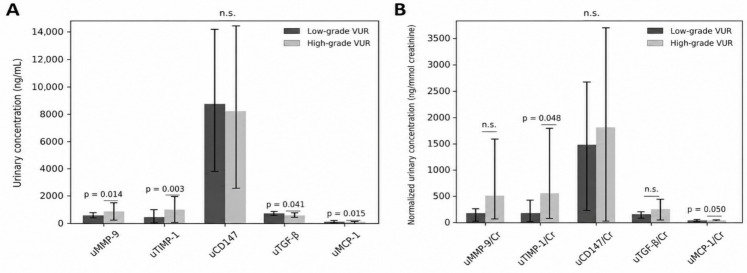
Urinary biomarkers according to VUR grade. (**A**) Absolute urinary concentrations of MMP-9, TIMP-1, CD147, TGF-β, and MCP-1 in patients with low-grade and high-grade vesicoureteral reflux (VUR). (**B**) Creatinine-normalized urinary levels of MMP-9, TIMP-1, CD147, TGF-β, and MCP-1 according to VUR grade. Data are presented as mean ± standard deviation. Group comparisons were performed using the Mann–Whitney U test.

Among patients with VUR, 41 were classified as having high-grade VUR, while 31 had low-grade VUR. Serum concentrations of CRP, urea, creatinine, MMP-9, TIMP-1, CD147, TGF-β, and MCP-1 did not differ significantly between patients with low-grade and high-grade VUR (*p* > 0.05). In urine samples, patients with high-grade VUR exhibited significantly higher levels of MMP-9 (*p* = 0.014), TIMP-1 (*p* = 0.003), and MCP-1 (*p* = 0.015) compared with those with low-grade VUR. Urinary TGF-β levels were significantly lower in the high-grade VUR group (*p* = 0.041). No significant differences were observed in urinary CD147 or urinary creatinine between the groups (*p* > 0.05). After normalization to urinary creatinine, uTIMP-1/Cr values were significantly higher in patients with high-grade VUR compared with those with low-grade VUR (*p* = 0.048). No significant differences were detected for uMMP-9/Cr, uCD147/Cr, TGF-β/Cr, or uMCP-1/Cr between the groups (*p* > 0.05), although uMCP-1/Cr showed a borderline difference (*p* = 0.050; Figure 2).

### 2.5. Serum and Urinary Biomarkers in Relation to Unilateral and Bilateral Renal Scarring

**Figure 3:**
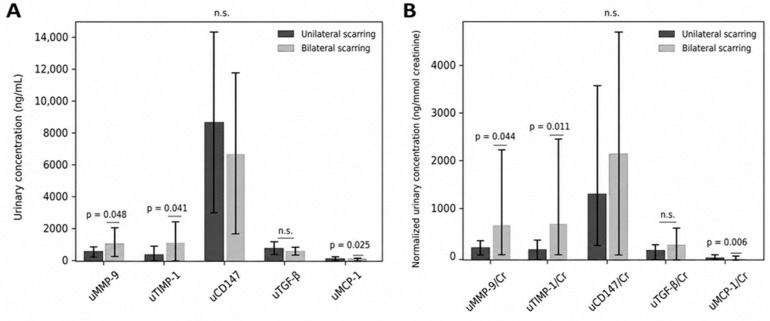
Urinary biomarkers according to renal scarring pattern. (**A**) Absolute urinary concentrations of MMP-9, TIMP-1, CD147, TGF-β, and MCP-1 in patients with unilateral and bilateral renal scarring. (**B**) Creatinine-normalized urinary levels of MMP-9, TIMP-1, CD147, TGF-β, and MCP-1 according to renal scarring pattern. Data are presented as mean ± standard deviation. Group comparisons were performed using the Mann–Whitney U test.

Serum concentrations of CRP, urea, creatinine, MMP-9, TIMP-1, CD147, TGF-β, and MCP-1 did not differ significantly between patients with unilateral and bilateral renal scarring (*p* > 0.05). Patients with bilateral renal scarring exhibited significantly lower urinary creatinine levels (*p* = 0.015) and significantly higher urinary levels of MMP-9 (*p* = 0.048), TIMP-1 (*p* = 0.041), and MCP-1 (*p* = 0.025) compared to patients with unilateral renal scarring. No significant differences were observed for urinary CD147 or urinary TGF-β between the groups (*p* > 0.05). After normalization to urinary creatinine, uMMP-9/Cr (*p* = 0.044), uTIMP-1/Cr (*p* = 0.011), and uMCP-1/Cr (*p* = 0.006) were significantly higher in patients with bilateral renal scarring compared with those with unilateral scarring. No significant differences were found for uCD147/Cr or sTGF-β/Cr between the groups (*p* > 0.05; Figure 3).

## 3. Discussion

Reflux nephropathy represents a complex pathophysiological process in which inflammation, ECM remodeling, and progressive fibrosis lead to permanent scarring of the renal parenchyma [15]. Traditional laboratory parameters, such as serum creatinine and urea, often remain within reference ranges in the early stages of the disease, as a significant decline in glomerular filtration occurs only after substantial loss of functional renal parenchyma. Therefore, identifying more sensitive biomarkers that reflect early molecular changes in the kidney is of major clinical importance [9]. In the past decade, increasing attention has been directed toward urinary biomarkers of inflammation and ECM remodeling, as they more directly reflect local processes within renal tissue [13]. In contrast to serum biomarkers, which represent the systemic response of the organism, urinary biomarkers originate directly from the renal parenchyma and urinary tract and therefore may more accurately reflect intrarenal pathological processes [16].

Our study evaluated serum and urinary levels of ECM-related biomarkers in children with VUR, aiming to assess their role in the development of reflux nephropathy.

When analyzing serum biomarker levels, we observed that only MCP-1 levels were significantly higher in the VUR group compared to healthy controls, while none of the examined markers showed significant variation according to VUR grade or the extent of renal scarring. The absence of differences in serum biomarker levels suggests that fibrotic signaling in early VUR may remain confined to the kidney, without systemic “spillover.” Supporting this, the study by Taranta-Janusz et al. demonstrated that serum concentrations of MMP-2, MMP-9, TIMP-1, and TIMP-2 did not differ significantly between VUR patients and controls [17]. A similar dissociation between local and circulating ECM mediators has been described in the early stages of chronic kidney disease and in models of obstructive nephropathy [5,18]. An additional factor that should be considered when interpreting our findings is the significant age difference between the VUR and control groups. Age-related variations in immune response and renal physiology may influence biomarker levels, and therefore, the observed differences should be interpreted with caution.

Regarding disease severity, our findings indicate that serum biomarkers do not reflect the anatomical severity of VUR or the extent of renal scarring. Similar observations have been reported by other authors, emphasizing that the pathophysiology of reflux nephropathy is primarily driven by local intrarenal processes, while systemic inflammatory responses may be absent [5]. The isolated increase in systemic MCP-1 observed in our study, in the absence of significant changes in other circulating ECM markers, may reflect early inflammatory activation preceding detectable systemic fibrotic remodeling. MCP-1 is a key chemokine mediating monocyte/macrophage recruitment to sites of renal injury and plays a crucial role in tubulointerstitial inflammation and fibrosis [4,6]. Several clinical studies investigating MCP-1 in children with VUR suggest that this chemokine reflects intrarenal inflammatory activity and may be useful in assessing the risk of renal scarring. In a prospective study, Pastore and Bartoli analyzed urinary MCP-1 excretion in children with moderate-to-severe primary VUR before and after endoscopic correction. They demonstrated significantly elevated urinary MCP-1 levels before intervention compared to controls, confirming active intrarenal inflammation. Following successful correction, an early decrease in MCP-1 levels was observed, indicating reduced acute inflammation. However, during follow-up, MCP-1 levels remained higher than in controls despite radiological correction, suggesting persistence of intrarenal inflammation. In the context of reflux nephropathy, persistent MCP-1 elevation may reflect ongoing inflammatory–fibrotic activity contributing to ECM remodeling and progressive renal scarring [19]. Similar findings were reported by Morozova et al., who analyzed urinary biomarkers of latent inflammation and fibrosis in children with different VUR grades. Urinary MCP-1 levels were highest in patients with severe reflux and did not decrease as expected six months after correction; in some subgroups, they even increased despite clinical improvement. DMSA scintigraphy and histological findings showed no significant improvement in existing scars. These results support the concept of “latent” or subclinical inflammation persisting after reflux correction [10]. Our findings align with this framework, as urinary MCP-1 levels were significantly higher in patients with high-grade VUR and in those with more extensive renal scarring.

Similar conclusions were drawn from the analysis of urinary MMP-9 and TIMP-1 levels, which were significantly elevated in higher grades of reflux and in cases of more extensive scarring. MMP-9 is a key proteolytic enzyme that degrades ECM components, particularly type IV collagen and gelatin, while TIMP-1 is its primary inhibitor, regulating the balance between ECM degradation and accumulation. Yilmaz et al. demonstrated that urinary MMP-9 and TIMP-1 levels were significantly higher in patients with renal scarring compared to those without, with MMP-9 showing high diagnostic accuracy for predicting scarring [20]. Similarly, studies in children with acute pyelonephritis have shown that elevated urinary MMP-9 levels are associated with subsequent renal scarring on DMSA scintigraphy [21]. The study by Taranta-Janusz et al. further demonstrated altered concentrations of MMP-2, MMP-9, TIMP-1, and TIMP-2 in serum and urine in children with VUR, particularly in higher grades of reflux. A relative predominance of TIMPs over MMPs and a reduced MMP/TIMP ratio were observed in grades III–V VUR, suggesting impaired ECM degradation and enhanced fibrogenesis [17].

Among ECM biomarkers, transforming growth factor beta (TGF-β) represents a central regulator of renal fibrogenesis. Its activation leads to fibroblast proliferation and increased collagen synthesis. MMP-9 can indirectly modulate TGF-β activity by releasing latent TGF-β from the ECM, thereby contributing to fibrosis progression [22]. One of the key studies in this context is that of Morozova et al., which showed that urinary TGF-β1 levels may remain elevated even after successful reflux correction, indicating persistent intrarenal fibrotic activity [10]. Similarly, Makieieva et al. demonstrated significantly higher urinary TGF-β1 levels in children with VUR, particularly in those with renal scars confirmed by DMSA scintigraphy. ROC analysis in that study showed excellent diagnostic performance of urinary TGF-β (AUC ≈ 0.90), suggesting its potential value in identifying renal scarring [23]. In our study, creatinine-normalized urinary TGF-β was also significantly higher in VUR patients compared to controls, supporting the concept of early intrarenal production and urinary excretion. ROC curve analysis further demonstrated that uTGF-β/Cr and uTIMP-1/Cr showed moderate diagnostic accuracy for discriminating patients with VUR from controls (AUCs of 0.73 and 0.74, respectively).

Although the observed AUC values indicate moderate diagnostic accuracy, these biomarkers alone are unlikely to be sufficient for clinical decision-making. Instead, they may serve as complementary tools alongside established diagnostic methods.

The discrepancy between absolute urinary biomarker concentrations and creatinine-normalized values observed in our study highlights the importance of correcting for urine dilution. Variations in urinary concentration, particularly in pediatric populations, may obscure true biological differences when absolute values are considered alone. Normalization to urinary creatinine provides a more reliable estimation of biomarker excretion and may better reflect intrarenal pathological processes. This finding underscores the necessity of interpreting urinary biomarkers in the context of creatinine adjustment.

However, we also observed significantly lower urinary TGF-β levels in high-grade VUR, which may appear paradoxical. This finding can be explained by stage-dependent dynamics of fibrogenesis. In the early stages, increased production leads to higher urinary excretion, whereas in advanced disease, TGF-β becomes sequestered within fibrotic tissue, and tubular damage reduces its urinary release. Despite lower urinary TGF-β levels in severe VUR, ROC analysis demonstrated that the uTGF-β/Cr ratio retains diagnostic value. This suggests that biomarker performance depends more on its ability to distinguish pathological from physiological states than on linear increases with disease severity.

CD147 (basigin) has recently gained attention as a regulator of MMP expression. It stimulates MMP-9 production and enhances ECM remodeling and inflammatory cell migration [24]. Although no clinical studies have directly investigated CD147 in children with VUR, experimental and clinical evidence from other renal diseases suggest its involvement in fibrosis. In a unilateral ureteral obstruction model, basigin-deficient mice exhibited significantly reduced renal fibrosis, indicating its active role in ECM remodeling [25]. Clinical studies in IgA nephropathy and diabetic nephropathy have shown that plasma CD147 correlates with renal function and histological damage [26], while elevated urinary CD147 has been reported in kidney transplant rejection [27]. Interestingly, urinary CD147 levels were significantly lower in our VUR cohort, suggesting complex stage-dependent regulation. The observed reduction in urinary CD147 levels in patients with VUR may appear counterintuitive, given its known role as an inducer of MMP expression and promoter of ECM remodeling [28]. One possible explanation lies in the stage-dependent dynamics of renal injury, where CD147 may be retained within the renal tissue during active fibrosis, resulting in reduced urinary excretion. Alternatively, tubular epithelial damage may impair its release into the urine. It is also possible that CD147 exhibits differential regulation compared to other ECM-related mediators, reflecting a more complex and not yet fully understood role in reflux nephropathy. Although CD147 has been implicated in renal fibrosis, its role in VUR remains unclear. In the present study, the observed changes in urinary CD147 should be interpreted cautiously, as no direct mechanistic evidence supports its involvement in VUR. Therefore, CD147 should be considered a promising but exploratory biomarker requiring further validation. Recent studies suggest that while individual biomarkers have limited diagnostic performance, their combination into panels significantly improves predictive accuracy [29,30].

This supports a multimarker approach incorporating inflammation, ECM remodeling, and fibrogenesis pathways.

The multivariate analysis performed for the primary comparison between patients with VUR and controls did not confirm independent associations between urinary biomarkers and VUR after adjustment for age and sex. This finding suggests that the differences observed in univariate analyses may be partially influenced by baseline group characteristics, particularly age. Importantly, the direction of associations remained consistent between univariate and multivariate analyses, supporting the internal validity of the findings despite attenuation of statistical significance after adjustment. The significant effect of age in the adjusted model further highlights its role as a potential confounder, which is consistent with the observed age imbalance between the study groups. These results underscore the importance of considering confounding variables in pediatric biomarker studies and suggest that the observed associations should be interpreted as exploratory and hypothesis-generating. Further studies with larger, age-balanced cohorts are required to clarify the independent role of these biomarkers.

Our findings support the concept that early VUR is characterized by localized intrarenal processes, which may explain why changes are more pronounced in urinary than in serum biomarkers. This dissociation highlights the potential of urinary biomarkers as early, non-invasive indicators of renal injury. Combined biomarker analysis may enable improved identification of patients at risk for reflux nephropathy and more precise monitoring of disease progression. Such an approach could complement existing imaging modalities, such as DMSA scintigraphy.

Several limitations of this study should be acknowledged. First, the relatively small sample size and the single-center design may limit the generalizability of the findings. Second, a significant age difference between the VUR and control groups represents an important limitation and may have influenced biomarker levels in the primary comparative analyses. Although multivariate analyses adjusted for age and sex were performed, residual confounding cannot be excluded. In addition, other potential clinical confounders, such as prior urinary tract infections, medication use, and individual variability in inflammatory status, were not specifically addressed in the present analyses. Furthermore, the cross-sectional design precludes assessment of causal relationships and does not allow evaluation of the prognostic value of these biomarkers over time. The cut-off values identified in this study should be interpreted with caution, as no internal or external validation was performed. Given the moderate diagnostic performance observed, these thresholds should be considered preliminary and require validation in independent cohorts before clinical application. Finally, although urinary biomarkers were normalized to creatinine, variability in urine concentration and collection conditions may still have affected the results. Future longitudinal and multicenter studies with larger cohorts are needed to validate these findings and to further define the clinical utility of ECM-related biomarkers in VUR.

In conclusion, this study demonstrates that urinary, rather than serum, ECM-related biomarkers more accurately reflect intrarenal inflammatory and fibrotic processes in children with vesicoureteral reflux. Among the investigated markers, creatinine-normalized TIMP-1 showed consistent associations with disease severity, suggesting a potential role of MMP/TIMP imbalance in the progression of renal injury. However, after adjustment for potential confounding factors, these associations were attenuated, indicating that the findings should be interpreted with caution. While individual biomarkers showed moderate diagnostic performance, their combined assessment may enhance risk stratification. However, the role of specific biomarkers, including CD147, remains to be further clarified. Overall, these findings support the potential value of urinary biomarkers as non-invasive indicators of renal involvement, but further validation in larger, longitudinal studies is required to confirm their clinical utility and to support their integration into personalized diagnostic and therapeutic approaches in children with VUR.

## 4. Materials and Methods

### 4.1. Subjects Selection

This study was designed as a cross-sectional case–control study including 72 pediatric patients with vesicoureteral reflux (VUR) and 21 healthy controls. The diagnosis of VUR was established by voiding cystourethrography (VCUG). Based on VCUG findings, VUR grades I–III were classified as low-grade reflux, while grades IV–V were considered high-grade reflux. Renal cortical scarring was assessed using technetium-99m dimercaptosuccinic acid (^99m^Tc-DMSA) renal scintigraphy, and it was defined as a persistent focal or diffuse cortical photon-deficient area associated with cortical thinning and/or contour deformity on delayed imaging performed at least 6 months after resolution of acute urinary tract infection. DMSA scintigraphy was interpreted by different physicians who were blinded to the patients’ clinical findings. According to DMSA findings, patients were further categorized into subgroups with unilateral renal scarring and those with bilateral renal scarring. All subjects were in a clinically stable phase at the time of sampling and had remained free of clinical signs of urinary tract infection for at least one month prior to sample collection. Exclusion criteria for patient inclusion comprised congenital anomalies of the kidney and urinary tract other than VUR, obstructive uropathies, known chronic kidney disease, acute infection at the time of sampling, systemic inflammatory or autoimmune diseases, malignancy, and prior renal surgery. Patients receiving immunosuppressive or long-term anti-inflammatory therapy were also excluded. Healthy controls were sex-matched children recruited during routine clinical evaluation, with no history of vesicoureteral reflux, urinary tract disease, recurrent urinary tract infections, or systemic inflammatory conditions. All control subjects underwent urinary tract ultrasound examination and showed no signs of urinary tract infection or structural urinary tract abnormalities at the time of evaluation. Although controls were selected to be comparable, a significant age difference between groups was observed. The study was conducted in accordance with the Declaration of Helsinki and was approved by the Ethics Committee of the University Clinical Center Niš (approval number 13235/7). Written informed consent was obtained from parents or legal guardians before participation.

### 4.2. Sample Collection and Biomarker Measurements

Blood and urine samples were collected under standardized conditions during routine clinical evaluation. All samples were obtained in the morning, at approximately the same time for all participants, in order to minimize potential diurnal variation. All subjects were in a clinically stable phase at the time of sampling, and none had evidence of acute urinary tract infection. Blood samples were obtained by venipuncture, allowed to clot at room temperature, and centrifuged at 3000× *g* for 10 min to separate serum. Serum aliquots were stored at −80 °C until analysis. Spot urine samples were collected at the same visit, centrifuged to remove cellular debris, aliquoted, and stored at −80 °C until further processing. All samples were thawed only once prior to analysis.

### 4.3. Serum Biomarker Determination

Serum concentrations of matrix metalloproteinase-9 (MMP-9), tissue inhibitor of metalloproteinases-1 (TIMP-1), CD147 (EMMPRIN), transforming growth factor-β (TGF-β), and monocyte chemoattractant protein-1 (MCP-1) were determined using commercially available Human Quantikine ELISA kits (R&D Systems, Minneapolis, MN, USA), according to the manufacturer’s instructions. Serum levels of MMP-9 and TIMP-1 were expressed in ng/mL, while serum concentrations of CD147, TGF-β, and MCP-1 were expressed in pg/mL, in accordance with the analytical characteristics and dynamic ranges specified by the manufacturer.

### 4.4. Urinary Biomarker Determination

Urinary concentrations of MMP-9, TIMP-1, and MCP-1 were measured using commercially available Human ELISA kits (Abcam, Cambridge, UK), following the manufacturer’s protocols. Urinary levels of TGF-β and CD147 were determined using Human Quantikine ELISA kits (R&D Systems, Minneapolis, MN, USA). All urinary biomarker concentrations were initially expressed in pg/mL.

### 4.5. Urinary Creatinine Determination and Normalization

Urinary creatinine concentrations were measured using a standard enzymatic colorimetric method on an automated clinical chemistry analyzer, according to the manufacturer’s instructions. To correct for urine dilution, urinary biomarker concentrations were normalized to urinary creatinine and expressed as ng/mmol creatinine. Creatinine-normalized values were used for comparative analyses and receiver operating characteristic (ROC) curve analyses where appropriate.

### 4.6. Quality Control

Calibration curves were generated for each analyte using recombinant standards provided by the manufacturers. The intra- and inter-assay coefficients of variation were within the limits specified by the manufacturers for all assays. The analytical sensitivity of the ELISA assays, expressed as the minimum detectable dose (MDD), varied with the analyte. For serum biomarkers, the MDD was 0.156 ng/mL for MMP-9 and 0.08 ng/mL for TIMP-1, while the minimum detectable concentrations for CD147, MCP-1, and TGF-β were 2.94 pg/mL, 1.7 pg/mL, and 7.0 pg/mL, respectively. For urinary biomarkers, the MDD was 10 pg/mL for MMP-9 and 40 pg/mL for TIMP-1, whereas the minimum detectable concentrations for MCP-1 and TGF-β were identical to those in serum.

### 4.7. Statistical Analysis

Statistical analysis was performed using IBM SPSS Statistics (version 20.0, SPSS Inc., Chicago, IL, USA). Continuous variables are presented as mean ± standard deviation (SD). Given the characteristics of the data and the relatively small sample size, differences between two independent groups were assessed using the Mann–Whitney U test. To control for multiple testing in the primary between-group biomarker comparisons, the Benjamini–Hochberg false discovery rate (FDR) correction was applied to the *p*-values reported in Table 2. Receiver operating characteristic (ROC) curve analysis was performed to evaluate the diagnostic performance of the investigated biomarkers and to assess their ability to discriminate between patients and controls. The area under the ROC curve (AUC), with corresponding 95% confidence intervals (CI), was calculated. Optimal cut-off values were determined using the Youden index, defined as the maximum sum of sensitivity and specificity. An a priori power analysis was performed using G*Power* software (version 3.1) to determine the required sample size. The calculation was based on a one-tailed Wilcoxon–Mann–Whitney test for two independent groups. With an effect size (Cohen’s d) of 1.46, a significance level (α) of 0.05, and a desired statistical power of 0.95, based on a pilot study, the required sample size was 12 participants per group (total *n* = 24). The achieved power was 0.96. Sample size calculation was based on the primary comparison between VUR patients and controls, using serum MCP-1 as the main variable of interest and assuming a large effect size. Additional analyses, including subgroup comparisons according to VUR severity and renal scarring pattern, were not specifically powered and were therefore considered exploratory. To assess the independent association between urinary biomarkers and vesicoureteral reflux (VUR), multivariate logistic regression analysis was performed. VUR status (0 = control, 1 = VUR) was used as the dependent variable. Separate models were constructed for each biomarker (uTIMP-1/Cr, uTGF-β/Cr, uCD147/Cr, and uMMP-9/Cr), adjusting for age and sex as potential confounding variables. Results were expressed as odds ratios (ORs) with 95% confidence intervals (CIs). A *p*-value < 0.05 was considered statistically significant.

## Figures and Tables

**Table 1:** Clinical and Demographic Characteristics of the Study Groups.

Parameter	VUR	Control Group	*p*-Value ^1^
Age (months)	88.31 ± 63.67	151.24 ± 36.79	<0.001
Sex, n (%)			1.000
Male	35 (48.6%)	10 (47.6%)	
Female	37 (51.4%)	11 (52.4%)	
CRP (mg/L)	4.38 ± 4.14	6.57 ± 2.30	0.001
Serum urea (mmol/L)	4.27 ± 1.30	3.61 ± 1.54	0.006
Serum creatinine (µmol/L)	52.17 ± 23.37	51.86 ± 10.60	0.298
Urinary microalbumin (mg/L)	37.59 ± 105.65	27.90 ± 42.23	0.777
Urinary creatinine (mmol/L)	7.22 ± 5.28	10.59 ± 4.90	0.006

^1^ Data are presented as mean ± standard deviation or number (percentage). *p*-values were calculated using the Mann–Whitney U test.

**Table 2:** Serum and Urinary Biomarker Levels in Patients and Controls.

Biomarker	VUR	Control Group	*p*-Value ^1^	Adjusted *p*
**Serum**				
MMP-9 (ng/mL)	172.33 ± 87.16	190.8 ± 85.45	0.401	0.501
TIMP-1 (ng/mL)	274.33 ± 107.52	268.6 ± 164.08	0.296	0.404
CD147 (pg/mL)	2635.32 ± 1149.17	2171.72 ± 870.03	0.174	0.326
TGF-β (pg/mL)	37,267.48 ± 10,724.67	37,876.64 ± 5632.66	0.562	0.602
MCP-1 (pg/mL)	284.80 ± 193.70	73.86 ± 67.07	<0.001	0.004
**Urine**				
uMMP-9 (pg/mL)	670.12 ± 494.16	668.8 ± 228.49	0.170	0.326
uTIMP-1 (pg/mL)	603.99 ± 1037.01	317.17 ± 410.18	0.520	0.600
uCD147 (pg/mL)	8371.19 ± 5387.41	22,043.69 ± 8112.99	0.000	0.004
uTGF-β (pg/mL)	543.58 ± 149.48	545.34 ± 148.77	0.796	0.796
uMCP-1 (pg/mL)	14.95 ± 9.23	23.25 ± 21.42	0.261	0.404
**Urine** **(Biomarker/Creatinine ratio)**				
uMMP-9/Cr (ng/mmol)	280.21 ± 865.78	71.31 ± 68.98	0.091	0.228
uTIMP-1/Cr (ng/mmol)	277.07 ± 1007.59	46.77 ± 97.11	0.006	0.030
uCD147/Cr (ng/mmol)	1561.24 ± 1666.11	2053.13 ± 1347.08	0.011	0.033
uTGF-β/Cr (ng/mmol)	142.36 ± 179.37	55.89 ± 40.55	0.008	0.030
uMCP-1/Cr (ng/mmol)	5.15 ± 13.67	3.15 ± 5.04	0.277	0.404

^1^ Data are presented as mean ± standard deviation or number (percentage). *p*-values were calculated using the Mann–Whitney U test. To control for multiple testing, the Benjamini–Hochberg false discovery rate (FDR) correction was applied.

**Table 3:** Multivariate logistic regression analysis of urinary biomarkers associated with VUR.

Variable	OR	95% CI	*p*-Value
uMMP-9/Cr	1.004	0.997–1.011	0.297
uTIMP-1/Cr	1.003	0.997–1.010	0.310
uTGF-β/Cr	1.011	0.995–1.028	0.184
uCD147/Cr	0.9997	0.9992–1.0001	0.122

OR: odds ratio; CI: confidence interval. Multivariate logistic regression analysis was performed with VUR status as the dependent variable (0 = control, 1 = VUR), adjusting for age and sex. Separate models were constructed for each urinary biomarker.

## Data Availability

The data supporting the findings of this study are available within the article. Further inquiries may be directed to the corresponding author.

## Supplementary Materials

The following supporting information can be downloaded at: https://www.mdpi.com/article/10.3390/ijms27114815/s1.

## References

[1] Skoog S.J., Peters C.A., Arant B.S., Copp H.L., Elder J.S., Hudson R.G., Khoury A.E., Lorenzo A.J., Pohl H.G., Shapiro E. (2010). Pediatric vesicoureteral reflux guidelines panel summary report: Clinical practice guidelines for screening siblings of children with vesicoureteral reflux and neonates/infants with prenatal hydronephrosis. J. Urol..

[2] Mattoo T.K. (2011). Vesicoureteral reflux and reflux nephropathy. Adv. Chronic Kidney Dis..

[3] Lorenzo A.J. (2022). Vesicoureteral reflux, renal scars, and urinary tract infections in children: A new way to think about an old problem. Eur. Urol..

[4] Eddy A.A. (2000). Molecular basis of renal fibrosis. Pediatr. Nephrol..

[5] Chevalier R.L. (2006). Pathogenesis of renal injury in obstructive uropathy. Curr. Opin. Pediatr..

[6] Duffield J.S. (2014). Cellular and molecular mechanisms in kidney fibrosis. J. Clin. Investig..

[7] Liu Y. (2011). Cellular and molecular mechanisms of renal fibrosis. Nat. Rev. Nephrol..

[8] Remuzzi G., Benigni A., Remuzzi A. (2006). Mechanisms of progression and regression of renal lesions of chronic nephropathies and diabetes. J. Clin. Investig..

[9] Putri U.M.A., Raharja P.A.R., Situmorang G.R., Wahyudi I., Rodjani A., Puspitasari H.A., Imam A., Saraiva L.R., Vallasciani S., Abbas T.O. (2025). Biomarker for renal scarring screening in children with vesicoureteral reflux: A systematic review. Front. Pediatr..

[10] Morozova O., Morozov D., Pervouchine D., Einav Y., Lakomova D., Zakharova N., Severgina L., Maltseva L., Budnik I. (2020). Urinary biomarkers of latent inflammation and fibrosis in children with vesicoureteral reflux. Int. Urol. Nephrol..

[11] Tang P.M., Zhang Y.Y., Mak T.S., Tang P.C., Huang X.R., Lan H.Y. (2018). Transforming growth factor-β signalling in renal fibrosis: From Smads to non-coding RNAs. J. Physiol..

[12] Isaka Y. (2018). Targeting TGF-β signaling in kidney fibrosis. Int. J. Mol. Sci..

[13] de Oliveira Tavares J.O., Passos C.L., de Souza M.B.M., Leite M.T.C., Freitas L.G.F. (2026). Diagnostic accuracy of serum and urinary biomarkers for renal scarring in children with vesicoureteral reflux: A systematic review and meta-analysis. J. Pediatr. Surg..

[14] Colceriu M.C., Aldea P.L., Boț A.L., Bulată B., Delean D., Grama A., Mititelu A., Decea R.M., Sevastre-Berghian A., Clichici S. (2023). The utility of noninvasive urinary biomarkers for the evaluation of vesicoureteral reflux in children. Int. J. Mol. Sci..

[15] Li L., Fu H., Liu Y. (2022). The fibrogenic niche in kidney fibrosis: Components and mechanisms. Nat. Rev. Nephrol..

[16] Bieniaś B., Sikora P. (2020). Selected metal matrix metalloproteinases and tissue inhibitors of metalloproteinases as potential biomarkers for tubulointerstitial fibrosis in children with unilateral hydronephrosis. Dis. Markers.

[17] Taranta-Janusz K., Wasilewska A., Debek W. (2010). Serum and urinary concentration of metalloproteinases and their tissue inhibitors in children with high grade vesicoureteral reflux. Pediatr. Nephrol..

[18] Chevalier R.L., Forbes M.S., Thornhill B.A. (2009). Ureteral obstruction as a model of renal interstitial fibrosis and obstructive nephropathy. Kidney Int..

[19] Pastore V., Bartoli F. (2017). Urinary excretion of epidermal growth factor and monocyte chemoattractant protein-1 in children with vesicoureteral reflux. Int. Braz. J. Urol..

[20] Yilmaz A., Bilge I., Kiyak A., Gedikbasi A., Sucu A., Aksu B., Emre S., Sirin A. (2012). Matrix metalloproteinase-9 and tissue inhibitor of metalloproteinase-1 in vesicoureteral reflux. Pediatr. Nephrol..

[21] Abedi S.M., Mohammadjafari H., Rafiei A., Bazi S., Yazdani P. (2017). Urinary matrix metalloproteinase 9 and tissue inhibitor of metalloproteinase 1 biomarkers for predicting renal scar in children with urinary tract infection. Turk. J. Urol..

[22] Meng X.M., Nikolic-Paterson D.J., Lan H.Y. (2016). TGF-β: The master regulator of fibrosis. Nat. Rev. Nephrol..

[23] Makieieva N.I., Morozova O.O., Iarova K.K., Pryima Y.S., Golovachova V.O., Vygivska L.A. (2020). Urinary excretion of transforming growth factor beta-1 and vascular endothelial growth factor in children with vesicoureteral reflux. Wiad. Lek..

[24] Cheng Z., Zhang X., Zhang Y., Li L., Chen P. (2022). Role of MMP-2 and CD147 in kidney fibrosis. Open Life Sci..

[25] Kato N., Kosugi T., Sato W., Ishimoto T., Kojima H., Sato Y., Sakamoto K., Maruyama S., Yuzawa Y., Matsuo S. (2011). Basigin/CD147 promotes renal fibrosis after unilateral ureteral obstruction. Am. J. Pathol..

[26] Mori Y., Masuda T., Kosugi T., Yoshioka T., Hori M., Nagaya H., Maeda K., Sato Y., Kojima H., Kato N. (2018). The clinical relevance of plasma CD147/basigin in biopsy-proven kidney diseases. Clin. Exp. Nephrol..

[27] Futamura K., Tsujita M., Kosugi T., Ryuge A., Okada M., Hiramitsu T., Narumi S., Takeda A., Watarai Y., Morozumi K. (2025). Urinary basigin/CD147 is a useful marker of acute T cell-mediated rejection in kidney transplant recipients. Ren. Fail..

[28] Grass G.D., Toole B.P. (2015). How, with whom and when: An overview of CD147-mediated regulatory networks influencing matrix metalloproteinase activity. Biosci. Rep..

[29] Valério F.C., Lemos R.D., Reis A.L.d.C., Pimenta L.P., Vieira É.L., Silva A.C.E. (2020). Biomarkers in vesicoureteral reflux: An overview. Biomark. Med..

[30] Ganapathy S., Harichandrakumar K.T., Jindal B., Naik P.S., Nair N.S. (2023). Comparison of diagnostic accuracy of models combining renal biomarkers in predicting renal scarring in pediatric population with vesicoureteral reflux (VUR). Ir. J. Med. Sci..

