# Supplementary material for: Inflammation–Matrix Crosstalk in Vesicoureteral Reflux: Serum and Urinary ECM-Related Biomarkers and Renal Scarring
Source: Int J Mol Sci. 2026 May 27;27(11):4815. doi: 10.3390/ijms27114815 (PMC13257324; doi:10.3390/ijms27114815)
Supplement: Supplementary file 1 [file ijms-27-04815-s001.zip › ijms-4294590-supplementary.pdf]

Supplementary Table S1. Diagnostic performance of creatinine-normalized urinary biomarkers for VUR

Biomarker	AUC (95% CI)	Cut-off	Sensitivity (%)	Specificity (%)	*p*-value
uMMP-9/Cr	0.65 (0.49–0.80)	67.22	56.0	85.7	0.058
uTIMP-1/Cr	0.74 (0.61–0.87)	18.96	72.0	78.6	<0.001
uCD147/Cr†	0.72 (0.58–0.86)	≤1269.0	58.0	85.7	0.002
uTGF-β/Cr	0.73 (0.60–0.87)	45.88	76.0	64.3	<0.001

† *For uCD147/Cr, ROC analysis was performed after inversion of values to account for lower concentrations observed in patients with VUR.*
